# Sterilization of hydrogen peroxide resistant bacterial spores with stabilized chlorine dioxide

**DOI:** 10.1186/s13568-015-0109-4

**Published:** 2015-04-17

**Authors:** Anthony Friedline, Malcolm Zachariah, Amy Middaugh, Matt Heiser, Neeraj Khanna, Parag Vaishampayan, Charles V Rice

**Affiliations:** Department of Chemistry and Biochemistry, Stephenson Life Sciences Research Center, University of Oklahoma, Norman, Oklahoma USA; Bio-Cide International, Inc., Norman, Oklahoma USA; Jet Propulsion Laboratory, California Institute of Technology, Biotechnology and Planetary Protection Group, Pasadena, California USA

**Keywords:** *Bacillus pumilus*, *Bacillus subtilis*, Chlorine dioxide, Spore killing, Spores

## Abstract

*Bacillus pumilus* SAFR-032 spores isolated from a clean room environment are known to exhibit enhanced resistance to peroxide, desiccation, UV radiation and chemical disinfection than other spore-forming bacteria. The survival of *B. pumilus* SAFR-032 spores to standard clean room sterilization practices requires development of more stringent disinfection agents. Here, we report the effects of a stabilized chlorine dioxide-based biocidal agent against spores of *B. pumilus* SAFR-032 and *Bacillus subtilis* ATCC 6051. Viability was determined via CFU measurement after exposure. Chlorine dioxide demonstrated efficacy towards sterilization of spores of *B. pumilus* SAFR-032 equivalent or better than exposure to hydrogen peroxide. These results indicate efficacy of chlorine dioxide delivered through a stabilized chlorine dioxide product as a means of sterilization of peroxide- and UV-resistant spores.

**Electronic supplementary material:**

The online version of this article (doi:10.1186/s13568-015-0109-4) contains supplementary material, which is available to authorized users.

## Introduction

During periods of environmental stress or nutrient depletion, certain Gram-positive bacteria use sporulation to protect its DNA within a protective shell. Germination into a vegetative cell occurs when conditions improve. These processes of sporulation and germination are well understood and reviewed in relevant literature (Errington 1993; Driks 2002; Setlow 2003; Piggot and Hilbert 2004; Paredes-Sabja et al. 2011; Higgins and Dworkin 2012). While *Bacillus subtilis* is studied as a model organism for sporulation, other species are also relevant due to healthcare or infectious disease reasons, such as *Bacillus anthracis* (Schmid and Kaufmann 2002; Spotts Whitney et al. 2003), *Clostridium botulinum* (Chaudhry 2011), and *Clostridium difficile* (Roberts et al. 2008; Best et al. 2010; Peniche et al. 2013). Each layer of the spore structure protects against DNA damage. The outer layer, the coat, is laminar, serving as a permeation barrier against lysozymes and other agents (Riesenman and Nicholson 2000; Takamatsu and Watabe 2002; Driks 2004). The cortex is a cross linked peptidoglycan structure surrounding the core, implicated in heat resistance of the spore (Mallidis and Scholefield 1987; Atrih and Foster 1999); it differs from that found in vegetative cells (Warth and Strominger 1971; Warth and Strominger 1972) by converting half of its *N*-acetyl-muramic acids into muramic lactams, which are targets for cortex lysis during germination (Atrih et al. 1996). The spore core houses the bacterial DNA, small acid-soluble spore proteins (SASPs) (Setlow 1988; Moeller et al. 2009), and a calcium-dipicolinic acid chelate (Setlow et al. 2006; Magge et al. 2008) which serve to protect the DNA from UV damage. These features, along with a lower water content in the spore core (Setlow 2006), allow spores to survive for long periods of time.

These features facilitate bacterial spores to be highly resistant to a wide variety of potentially lethal conditions (Setlow 2006; Nicholson et al. 2000). This has led to studies analyzing the possibility of survival of spores in extraterrestrial conditions, such as Martian regolith (Schuerger et al. 2003; Kerney and Schuerger 2011; Moeller et al. 2012). In 2004, Link *et al*. published a study discussing UV resistance of spores of *Bacillus pumilus* isolated from the Spacecraft Assembly Facility at the Jet Propulsion Laboratory (JPL) in Pasadena, CA (Link et al. 2004). The isolates had higher UV resistance than a standard strain of *B. subtilis* used for dosimetry and other similarly-isolated *B. pumilus* strains. *B. pumilus* SAFR-032 was ~340 times more resistant to 254 nm UV radiation than the *B. pumilus* type strain, ATCC 7061, and was remarked to be “…the most highly UV-resistant spores yet to be discovered” (Link et al. 2004). *B. pumilus* SAFR-032 was reported as being the most resistant spore strain to UV radiation under simulated Martian environmental conditions the following year (Newcombe et al. 2005). *B. pumilus* SAFR-032 was also observed to have peroxide resistance (Link et al. 2004; Gioia et al. 2007). In 2012, Vaishampayan *et al.* noted that spores of *B. pumilus* SAFR-032 exhibited survival rates of 10-40% on spacecraft-grade aluminum coupons exposed to space vacuum for 18 months during the EXPOSE-E mission onboard the International Space Station (ISS) (Vaishampayan et al. 2012). Additionally, limited numbers of spores were able to germinate after exposure to solar UV radiation onboard the ISS, both exposed to space vacuum and within a simulated Martian environment. Given the known ability for habitable space vehicles, including the ISS, to harbor diverse and potentially hazardous bacteria (Venkateswaran et al. 2014), the risk of contamination by bacterial spores—and thus the need for a method of inactivating them—cannot be ruled out.

Chlorine dioxide, ClO_2_, is an oxidant known to exhibit antimicrobial properties (Beuchat et al. 2005; Gordon and Rosenblatt 2005; Novak et al. 2008; Rastogi et al. 2010). It has been demonstrated to have effects on many biological agents, such as *Bacillus anthracis* (Rastogi et al. 2010; Buhr et al. 2011), *Bacillus cereus* and *C. perfringens* (Foegeding et al. 1986), and *Legionella* sp. (Kim et al. 2002). It is unstable as a gas and is usually generated *in situ* for use, either electrochemically (Foegeding et al. 1986) or by reaction of sodium chlorite + chlorine gas, sodium hypochlorite + hydrogen chloride (Sharma and Sohn 2012), or sodium chlorate + peroxide and sulfuric acid (Gordon and Rosenblatt 2005). At sub-lethal concentrations, ClO_2_ may induce different behaviors in bacteria, with a study indicating *B. subtilis* formed biofilms at sub-lethal exposures to chlorine dioxide (Shemesh et al. 2010).

A 2003 paper examined the efficacy and potential mechanism of action of a stabilized chlorine dioxide solution against spores of *B. subtilis* compared with hypochlorite solution (Young and Setlow 2003). It was reported that there was no difference in efficacy between hypochlorite and chlorine dioxide, and that the mechanism does not damage DNA but likely damage to the internal membrane of the spore. These results were similar for a wild-type spore strain and a strain lacking the core-associated SASPs. The authors did state in their work that “…very little is known about the mechanisms of spore killing by and resistance to chlorine dioxide” (Young and Setlow 2003).

Given the known resistance of *B. pumilus* SAFR-032 to traditional sterilization methods, we investigated the efficacy of stabilized chlorine dioxide against spores of *B. pumilus* SAFR-032 and *B. subtilis* ATCC 6051. We report that chlorine dioxide is effective at killing spores of *B. subtilis* ATCC 6051 in a dose-dependent fashion. Further, we report the ability of chlorine dioxide to inactivate spores of the peroxide-resistant *B. pumilus* SAFR-032.

## Materials and methods

### Spore preparation

Frozen stocks of *B. pumilus* SAFR-032 (provided by NASA JPL) and wild-type *B. subtilis* ATCC 6051 (purchased from American Type Culture Collection, Manassas VA, US) were inoculated overnight in 20 mL of lysogeny broth (LB) media, which was used to inoculate 200 mL of media at 37°C in a 1 L Erlenmeyer flask. The *B. pumilus* SAFR-032 was grown solely in LB media until sporulation occurred due to nutrient exhaustion, which typically occurred after three days. *B. subtilis* ATCC 6051 was initially grown in LB media for one day. Cells were isolated with centrifugation (10,000 X *g*, 20 min, 4°C) and the supernatant decanted before suspension in an equal volume (200 ml) of chemically-defined sporulation medium (CDSM) (Hageman et al. 1984); the culture sporulated in CDSM over a period of 3-10 days. The different media used were based on assessment in our lab that *B. subtilis* sporulated more efficiently in CDSM, while *B. pumilus* SAFR-032 sporulated best in LB through nutrient exhaustion. Sporulation was monitored via phase-contrast microscopy of 5-10 μL wet mounts (Olympus BX-50) at a magnification of 1000X.

Once a desired level (>80%) of free spores were observed via phase contrast microscopy, spores were purified using a modification of the protocol of Zhao *et al.* (Zhao et al. 2008). Cultures were centrifuged at 10,000 x *g* for 20 minutes at 4°C; the supernatant was decanted and the pellet then resuspended in 200 mL of sterile Milli-Q water. The pellet was washed a second time with centrifugation (10,000 X *g*, 20 min, 4°C); after decanting, the pellet was resuspended in 20 mL of sterile 50% (v/v) EtOH:H_2_O in a centrifuge tube and allowed to shake (200 rpm) at 25°C for 2 hours to kill remaining vegetative cells. The suspension was spun down (10,000 X *g*, 20 min, 4°C), the supernatant decanted, and the pellet resuspended in 20 mL sterile Milli-Q water. The spores were washed by repeated (3x) centrifugation (10,000 X *g*, 20 min, 4°C) and resuspension in sterile Milli-Q water. After the final centrifugation and resuspension, the spore suspension was stored at 4°C overnight; the following morning the suspension was spun down, resuspended in sterile Milli-Q water, filtered through 3.1/1.2 μm glass fiber syringe filters connected in series, and stored at 4°C until used. The purified spore suspensions were then diluted with Milli-Q water to attain an optical density (OD_600_) of 0.01 (~10^6^ CFU mL^-1^), measured using a spectrophotometer (Beckman-Coulter DU 640). Spore preparations in glass test tubes were heat-shocked at 80°C for 15 min, followed by rapid cooling on ice to 4°C.

### Preparation of chlorine dioxide solutions

An aqueous solution of 2% stabilized chlorine dioxide (Oxine®; Bio-Cide International, Inc., Norman, OK, USA) was used in all exposure experiments. The product was activated by citric acid addition in a ratio of 0.2 g mL^-1^ of stabilized ClO_2_ solution without stirring (30 min, room temperature), causing the formation of chlorine dioxide. Each experiment used a freshly activated ClO_2_ solution. The total concentration of activated chlorine dioxide (after dilution) was measured using a proprietary iodometric titration method (Bio-Cide International). The concentration of free chlorine dioxide gas, which is a fraction of the total ClO_2_ concentration, was determined using a Hach DR3900 spectrophotometer (Hach Co., Loveland, CO) at λ = 445 nm. The activated ClO_2_ solution was then diluted in water to a total concentration of 500 μg/mL, followed by subsequent dilutions to approximately total concentrations of 200 μg/mL, 100 μg/mL, and 50 μg/mL for selected experiments. Experiments relying on a spray application of chlorine dioxide solution utilized 1 L of chosen dilutions in a hand-held pump-up sprayer (RL Flo-Master model 1401, Root-Lowell Manufacturing Co., Lowell, MI), which had been pressurized and flushed with at least 100 mL of the solution prior to use.

### Exposure of dried spores to chlorine dioxide

To evaluate inactivation of dried spores on glass cover slips, spore cultures were mixed (200 rpm) in 1:1 EtOH:H_2_O for 4 hours, and after the final centrifugation, the spore suspension was diluted to an OD_600_ of 1.0 (~10^8^ CFU mL^-1^). 100 μL of spore suspensions of both *B. subtilis* ATCC 6051 and *B. pumilus* SAFR-032 were each deposited onto three sterile microscope slide cover slips; these cover slips were allowed to dry at room temperature for 24 h in a laminar air flow chamber. After drying, the cover slips were exposed to a fine mist of stabilized chlorine dioxide solution at 47 or 187 μg/mL concentration for less than one second. The ClO_2_-treated samples were undisturbed for a desired duration (10 min, 60 min, or 24 h) in a laminar air flow chamber. Cover slips were transferred to a 50 mL Falcon® tube containing 10 mL 0.1 mol L^-1^ sodium thiosulfate to neutralize remaining active chlorine dioxide on the cover slips. The tubes containing the thiosulfate solution and cover slips were placed in an ultrasonic water bath (Ultrasonic Cleaner B-220, Branson Cleaning Equipment, Shelton, CT) for 2 minutes to dislodge the spores, followed by vigorous vortex mixing for 10 seconds (previously used in removal of *B. cereus* spores from metal coupons) (Tauveron et al. 2006). The resulting spore suspensions were serially diluted with sterile phosphate-buffered saline (PBS; 8 g L^-1^ NaCl, 0.2 g L^-1^KCl, 1.44 g L^-1^ Na_2_HPO_4_, 0.24 g L^-1^ KH_2_PO_4_, pH 7.4) and 100 μL portions were spread on TSA plates. Plates were incubated at 37°C to observe spore viability and the colonies counted after 48 h.

This procedure was also used with spores of *B. pumilus* SAFR-032 on spacecraft-grade aluminum coupons that had been autoclaved prior to use. The duration of exposure to chlorine dioxide was either 60 min or 24 h before neutralization. In addition to activated ClO_2_, coupons with dried *B. pumilus* SAFR-032 spores were also exposed to inactivated ClO_2_ (without citric acid) and 3.5% w/v hydrogen peroxide solutions for comparison of efficacy. A control set without exposure to any agent was also prepared. Two replicates (A and B) were prepared for both exposure durations. At the end of the exposure time, coupons were neutralized, sonicated, serially diluted and plated as described above; the resulting TSA plates were incubated at 37°C with colony counting performed 24 h after neutralization.

## Results

### Exposure of dried spores to chlorine dioxide

The exposure results of chlorine dioxide to dried spores on glass cover slips, both *B. pumilus* SAFR-032 and *B. subtilis* ATCC 6051, are shown in Table 1. As can be seen, chlorine dioxide is effective at inactivating spores of both species. This efficacy is dependent both on ClO_2_ concentration and duration of exposure. Inactivation is not complete at the lower (47 μg/mL) concentration, but it does increase with longer exposure durations. At the higher concentration (187 μg/mL), inactivation is much quicker, with no colonies having been detected on TSA plates even after a ten-minute exposure.

**Table 1:** **Viability assay of stabilized chlorine dioxide solution against spores of** ***B. subtilis*** **ATCC 6051 and** ***B. pumilus*** **SAFR-032**

		**Colony forming units**	
**ClO**_**2**_ **concentration (μg/mL)**	**Exposure time**	***B. subtilis*** **ATCC 6051**	***B. pumilus*** **SAFR-032**
0	N/A	4.59 ± 1.71 × 10^4^	1.30 ± 0.32 × 10^5^
47	10 m	4.70 ± 3.11 × 10^3^	2.40 ± 0.10 × 10^4^
47	60 m	3.50 ± 2.12 × 10^2^	1.10 ± 0.14 × 10^3^
47	24 h	n.d.	n.d.
187	10 m	n.d.	n.d.
187	60 m	n.d.	n.d.
187	24 h	n.d.	n.d.

Notes: Viability assay of *B. subtilis* ATCC 6051 and *B. pumilus* SAFR-032 dried spores on glass cover slips after exposure to stabilized chlorine dioxide solution at 47 or 187 μg/mL concentration and 10 min, 60 min, or 24 h exposure. n.d. = no colonies detected and thus CFU could not be determined.

Spores of *B. pumilus* SAFR-032 were dried onto sterile aluminum coupons and exposed to both active and inactive chlorine dioxide. Additional data was collected for the control sample and after exposure to 3.5% w/v hydrogen peroxide, an agent for which *B. pumilus* SAFR-032 has resistance (Link et al. 2004; Gioia et al. 2007). Table 2 shows the results of exposure of these agents against *B. pumilus* SAFR-032 spores at one- and 24-hour time intervals, as determined by CFU mL^-1^ values. These values were calculated from the raw plate counts after 24 h growth following exposure at each time interval, as shown in Additional file: 1 Tables S1 and S2 in the supporting information.

**Table 2:** **Counting of** ***B. pumilus*** **SAFR-032 spores (in CFU mL** ^**-1**^ **)**

**Exposure agent**	**CFU mL** ^**-1**^ **, 1 h exposure**	**CFU mL** ^**-1**^ **, 24 h exposure**
Nothing (control)	9.4 × 10^9^	2.65 × 10^9^
Peroxide, 3.5% v/v solution	1.47 × 10^8^	<5 × 10^2^
Inactivated ClO_2_ solution	2.79 × 10^8^	6.3 × 10^9^
Activated ClO_2_ solution	1.6 × 10^5^	<1 × 10^4^

Notes: Results of duplicate plate-counting experiments using 100 μL aliquots of exposed spores on aluminum coupons in serial dilutions of PBS following ultrasonication into sodium thiosulfate to inactivate chlorine dioxide. Inactive ClO_2_ concentration = 108.5 μg/mL total ClO_2_; active ClO_2_ concentration = 70.4 μg/mL total ClO_2_, 39 μg/mL free ClO_2_. CFU mL^-1^ values based on the first plates in the serial dilution to not be too numerous to count (>300 total CFU or >100 CFU in one quarter of plate).

At the shorter time interval, activated chlorine dioxide demonstrates a 4-log reduction in viability compared to the control. The presence of viable colonies after activated-ClO_2_ treatment is plausible given the ClO_2_ concentration measured (70.4 μg/mL) and detection of viable spores at a lower concentration as seen in Table 1. The activated stabilized-ClO_2_ clearly has sporicidal properties against *B. pumilus* SAFR-032; however, inactivated ClO_2_ has no measureable effect. Hydrogen peroxide treatment produces a one order-of-magnitude inactivation compared to the control, less effective than activated chlorine dioxide. At the longer duration of exposure, the peroxide and activated ClO_2_ are comparable in near-total spore kill. Inactivated chlorine dioxide still has no efficacy, being no better than the unexposed control. The almost-total lack of viable spores at the most concentrated solution shows that the efficacy of chlorine dioxide increases with longer durations, as previously seen in Table 1. Additionally, while 3.5% peroxide solution does show near-total inactivation at 24 hour exposure, the much-reduced inactivation in the one-hour exposure test indicates peroxide is slower-acting against *B. pumilus* SAFR-032 than chlorine dioxide.

## Discussion

### Efficacy of chlorine dioxide on spore inactivation, comparison with hydrogen peroxide

Stabilized chlorine dioxide could be useful for inactivation of resistant spores. As Table 1 shows, there is no appreciable loss of efficacy between *B. subtilis* ATCC 6051 and *B. pumilus* SAFR-032 inactivation at 47 μg/mL. At higher concentrations, inactivation is rapid enough that no viable colonies were found in plate counting after a 10 minute exposure.

When chlorine dioxide was compared against hydrogen peroxide, as shown in Table 2, it demonstrated a greater inactivation at the one-hour exposure time than peroxide against *B. pumilus* SAFR-032. Considering the known peroxide-resistance traits in this species (Link et al. 2004; Gioia et al. 2007), this data is not unexpected. The three-log difference in the CFU mL^-1^ value indicates that chlorine dioxide is able to circumvent the resistance properties of the spore and inactivate it in some manner. The chlorine dioxide must be activated, however; it is clear from the results that inactivated ClO_2_ has no measurable sporidical properties. Either inactive chlorine dioxide solution cannot permeate the spore, or chlorine dioxide is not activated within the spore environment. At the longer duration exposure of 24 hours, the chlorine dioxide and peroxide are roughly equal in sporicidal efficacy. The longer time necessary for peroxide to inactivate spores of *B. pumilus* SAFR-032 may result from a slow rate of penetration for peroxide, a slower rate of oxidative attack, or depletion of protective molecules in the dormant spore, such as peroxidases (Gioia et al. 2007; Checinska et al. 2012; Tirumalai et al. 2013). Another possibility arises from study of the oxidation potentials of the stabilized solution, which activated and in solution is primarily chlorine dioxide and chlorite (ClO_2_^-^), and hydrogen peroxide. While H_2_O_2_ has higher redox potential than ClO_2_ or ClO_2_^-^ (1.776 V vs. 0.954 V or 0.76 V), our results indicate less inactivation from the peroxide. It is possible that the different oxychloro species in the activated solution, mainly chlorine dioxide and chlorite, have a synergistic effect to enhance the overall sporicidal properties of the solution.

Despite the resistance mechanisms of *B. pumilus* SAFR-032 for peroxides, the chlorine dioxide treatment demonstrated efficacy against spores of this resistant strain as noted in Tables 1 and 2. The effects of ClO_2_ appear equally valid for *B. subtilis* ATCC 6051 based on the results of Table 1. The efficacy of chlorine dioxide against *B. pumilus* spores in general has not, to our knowledge, been studied before, and in particular not against *B. pumilus* SAFR-032. This work could be of importance for inactivation and sterilization of surfaces contaminated with *B. pumilus* SAFR-032 in particular, and *Bacillus* spores in general.

Additionally, this work could form the basis for novel clinical cleaning procedures for pathogenic spore-forming bacteria in hospital environments. By showing chlorine dioxide-based sterilization of bacterial spores, including UV-resistant species such as *B. pumilus* SAFR-032, activated chlorine dioxide applied as a liquid could be used in a clinical setting for disinfecting and sterilization of surfaces contaminated by dormant bacterial spores, as gaseous chlorine dioxide has been shown capable of performing (Lowe et al. 2013). This treatment could also be effective against contamination with *C. difficile* spores; such spores are only effectively neutralized with chlorite-containing hospital cleaning agents, as alcohol-based agents have no efficacy and chlorite-containing solutions could be harmful to hospital equipment in the concentrations necessary to achieve spore inactivation (MacLeod-Glover and Sadowski 2010; Gould and McDonald 2008). Stabilized chlorine dioxide solutions applied to contaminated surfaces could serve as a useful tool in eliminating pathogenic spores from clinical settings with less negative impacts on equipment or individuals than bleach or other chlorite-based cleaning agents.

## Additional file

Additional file 1: Table S1. Plate counting of *B. pumilus* SAFR-032 spores, one-hour exposure time. **Table S2.** Plate counting of *B. pumilus* SAFR-032 spores, 24-hour exposure time.
