# Supplementary material for: Sterilization of hydrogen peroxide resistant bacterial spores with stabilized chlorine dioxide
Source: AMB Express. 2015 Apr 17;5:24. doi: 10.1186/s13568-015-0109-4 (PMC4398677; doi:10.1186/s13568-015-0109-4)
Supplement: Additional file 1: Table S1. — Plate counting of B. pumilus SAFR-032 spores, one-hour exposure time. Table S2. Plate counting of B. pumilus SAFR-032 spores, 24-hour exposure time. [file 13568_2015_109_MOESM1_ESM.pdf]

Supplementary Information for the AMB Express Manuscript:

Sterilization of Hydrogen Peroxide Resistant Bacterial Spores with Stabilized Chlorine Dioxide

Anthony Friedline[1], Malcolm Zachariah[1], Amy Middaugh[1], Preston Larson[2], Greg Strout[2], Matt Heiser[3], Neeraj Khanna[3], Parag Vaishampayan[4], and Charles V. Rice[1#]

[1] Department of Chemistry and Biochemistry, Stephenson Life Sciences Research Center, University of Oklahoma, Norman, Oklahoma, USA; [2] Samuel Roberts Noble Microscopy Laboratory, George Lynn Cross Hall, University of Oklahoma, Norman, Oklahoma, USA; [3] Bio-Cide International, Inc., Norman, Oklahoma, USA; [4] Jet Propulsion Laboratory, California Institute of Technology, Biotechnology and Planetary Protection Group, Pasadena, California, USA

# Address correspondence to Prof. Charles V. Rice, rice@ou.edu.

Running headline: Spore killing by chlorine dioxide

TABLE S1 Plate counting of *B. pumilus* SAFR-032 spores, one-hour exposure time

		CFU (by serial dilution)†					
		10^{-1}	10^{-2}	10^{-3}	10^{-4}	10^{-5}	10^{-6}
Control	A	TNTC	TNTC	TNTC	TNTC	79	8
	B	TNTC	TNTC	TNTC	TNTC	109*	5
Peroxide, 3.5%	A	TNTC	TNTC	TNTC	181	6	2
	B	TNTC	TNTC	TNTC	113	5	2
Inactive Oxine	A	TNTC	TNTC	TNTC	298	27	7
	B	TNTC	TNTC	TNTC	260	16	0
Active Oxine	A	201	14	0	0	1	0
	B	TNTC	18	1	1	0	0*

Table S1. Plate counting of post-Oxine®-exposure *B. pumilus* SAFR-032 spores exposed on aluminum coupons using serial dilutions, on TSA plates. Exposure time to Oxine® was 1h; results shown are at 24h incubation time. Inactivated Oxine® concentration = 108.5 ppm ClO_2. Activated Oxine® concentration = 70.4 ppm total ClO_2, 39 ppm free ClO_2. A and B are duplicate platings. TNTC = too numerous to count (>300 colonies total or >100 colonies in one quarter of plate). * = plate appeared damaged or contaminated. † = '10^{-1}' represents plating of spores directly after ultrasonication (into 10 mL sodium thiosulfate, effective dilution 100:1); remaining dilutions are 10:1 dilutions into PBS.

TABLE S2 Plate counting of *B. pumilus* SAFR-032 spores, 24-hour exposure time

		CFU (by serial dilution) †					
		10^{-1}	10^{-2}	10^{-3}	10^{-4}	10^{-5}	10^{-6}
Control	A	TNTC	TNTC	TNTC	TNTC	31	2*
	B	TNTC	TNTC	TNTC	TNTC	20	2
Peroxide, 3.5%	A	0	0	0	0	0	0
	B	1	0	0	0	0	0
Inactive Oxine	A	TNTC	TNTC	TNTC	TNTC	48*	8
	B	TNTC	TNTC	TNTC	TNTC	78	3
Active Oxine	A	3	0	0	0	0	0
	B	3	0	0	0	0	0

Table S2. Plate counting of post-Oxine®-exposure *B. pumilus* SAFR-032 spores via serial dilution onto TSA plates. Oxine® concentrations are equal to those in Table S1. Exposure time was 24h with counting after 24h incubation. TNTC = too numerous to count (>300 colonies per plate or >100 colonies in one quarter of plate). * = agar plate appeared damaged or contaminated. † = ’10^{-1}’ represents plating of spores directly after ultrasonication (into 10 mL sodium thiosulfate, effective dilution 100:1); remaining dilutions are 10:1 dilutions into PBS.
